# Multiscale numerical simulation of in-plane mechanical properties of two-dimensional monolayers

**DOI:** 10.1039/d1ra01924d

**Published:** 2021-06-07

**Authors:** Sadegh Imani Yengejeh, Seyedeh Alieh Kazemi, William Wen, Yun Wang

**Affiliations:** Centre for Catalysis and Clean Energy, School of Environment and Science, Griffith University Gold Coast Campus QLD 4222 Australia yun.wang@griffith.edu.au

## Abstract

Many applications of two dimensional (2D) materials are often achieved through strain engineering, which is directly dependent on their in-plane mechanical characteristics. Therefore, understanding the in-plane mechanical characteristics of the 2D monolayers becomes imperative. Nevertheless, direct experimental measurements of in-plane mechanical properties of 2D monolayers face great difficulties due to the issues related to the availability of high-quality 2D materials and sophisticated facilities. As an alternative, numerical simulation has the potential to theoretically predict such properties. This review presents some recent progress in numerically exploring the in-plane mechanical properties of 2D materials, including first-principles density functional theory, force-field based classical molecular dynamics, and the finite-element method. The relevant case studies are provided to describe the applications of these methods along with their pros and cons. We hope that the multiscale simulation methods discussed in this review will inspire new ideas and boost further advances of the computational study on the in-plane mechanical properties of 2D materials.

## Abstract 2

The recent progress of multiscale numeric methods for investigating in-plane mechanical properties of 2D monolayers is reviewed.

## Graphical abstract

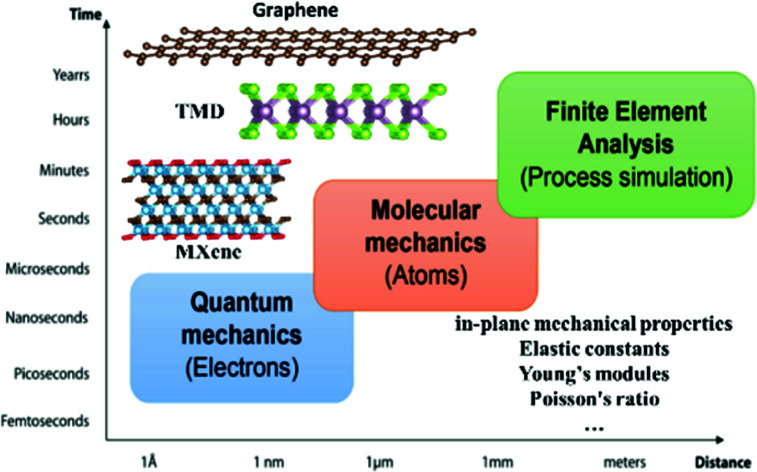

## 1. Introduction

**Fig. 1:**
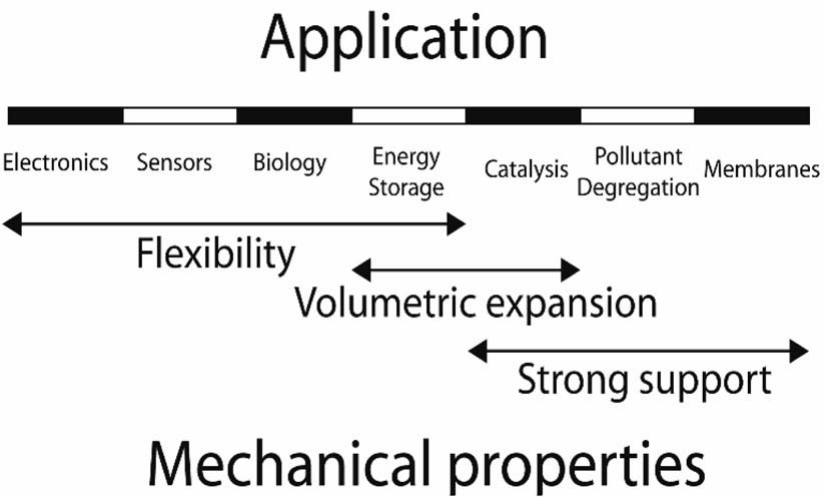
Potential applications of 2D materials related to their in-plane mechanical characteristics.

The discovery of graphene through the mechanical exfoliation of bulk graphite opens the field of two-dimensional (2D) materials.^1^ To date, many 2D materials including hexagonal boron-nitride (h-BN), phosphorene, MXene, layered double hydroxide (LDH), metal–organic frameworks (MOFs), covalent organic frameworks (COFs), and transition metal dichalcogenides (TMDs) have been experimentally and theoretically investigated.^2–29^ The mechanical properties of 2D materials have become a hot research topic because mechanical properties are physical features when a material is acted upon by external forces during the large-scale practical applications.^30^ 2D materials can be deformed by in-plane stretching or by out-of-plane bending. Therefore, the mechanical characteristics of 2D structures include both in-plane and bending moduli. For 2D monolayers, the in-plane mechanical properties have attracted specific attention since they are directly relevant to the applications through strain engineering, as illustrated in Fig. 1.^31–39^ As outlined, the 2D materials are promising candidates for stretchable transparent electrodes with high conductance and high flexibility.^40^ The resistance of the graphene electrode displayed no evident variation up to a bending radius of 2.3 mm corresponding to the uniaxial tensile strain of 6.5%. Meanwhile, the pre-strained substrates could enhance the bending limit up to ∼11%. 2D nanoelectromechanical systems (NEMS) resonators are strongly dependent on their mechanical properties, *e.g.* Young's modulus, mass density, and resonant frequency.^41^ The presence of piezoelectricity characteristic coupled with the mechanical flexibility of some 2D materials enables their promising applications in wearable power-generated nano-devices. The associated piezoresistive and piezoelectric effects under mechanical strain in 2D structures extend their applications to a diverse range in nano-industries.^42–45^ The 2D hybrid organic–inorganic perovskites (HOIPs) solar cells possess power-conversion efficiencies surpassing 25%.^46,47^ These 2D structures are promising applicants for the next generation of optoelectrical nano-devices, which bandgap can be tuned through strain engineering.^48,49^ Jiao *et al.* conducted a systematic study to investigate the structural and mechanical properties of TMDs using DFT calculations. Their theoretical studies revealed that the 2D TcS_2_ and TcSe_2_ monolayers exhibit promise as potential candidates for light harvesting.^50^ Furthermore, the analysis of the band alignment relative to the vacuum level showed that the TcSe_2_ monolayer is potentially plausible for water splitting. Meanwhile, a 2% compressive strain can also make the TcS_2_ monolayer suitable for photocatalysts. Considering the importance of strain engineering for 2D materials, many efforts have been devoted to exploring their in-plane mechanical characteristics. Table 1 lists the details of typical in-plane mechanical properties under the different external forces. The in-plane elastic moduli, Young's moduli, shear moduli, and Poisson's ratio are the parameters widely investigated in most studies.

**Table 1:** The description of the most important mechanical properties

Mechanical property	Description
Strength	The amount of the load that a material can tolerate before failure
Brittleness	The property of a material when the material fractures under stress without exhibiting much elastic deformation or changes in dimension
Stiffness	A material's ability to resist significant elastic deformation while loading
Hardness	A material's ability to resist various forms of deformation, indentation, and penetration
Toughness	A material's capacity to withstand elastic and plastic deformation without failure
Elasticity	A material's capacity to rebound back to their original dimensions after the deformation or being removed from its load
Anisotropy	The property difference in terms of the direction or orientation of the material
Ductility	A material's capacity to be stretched due to tensile stress
Creep	A slow and gradual deformation (or change in dimensions) of materials under a certain applied load in terms of time and temperature
Thermal expansion	A change in shape, volume or area caused by changes in temperature
Poisson's ratio	Poisson's ratio defines the ratio of transverse strain to the axial strain

**Fig. 2:**
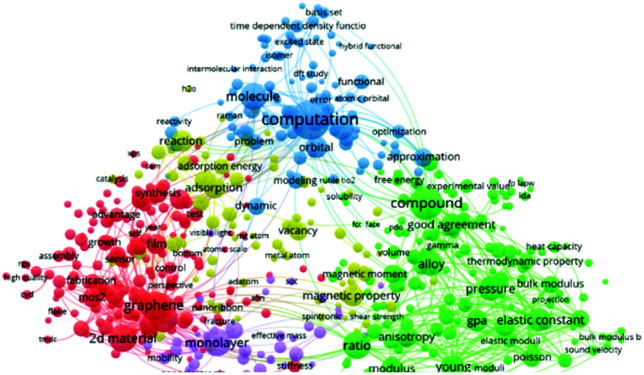
A visualized landscape of the recent studies regarding the mechanical characteristics of 2D materials.

There are different kinds of experimental technologies developed to measure the in-plane mechanical properties of 2D materials. These include (1) nanoindentation of suspended monolayer using atomic force microscopy (AFM); and (2) pressurized blister tests. Lee *et al.* first used the AFM nanoindentation method to investigate the mechanical characteristics of graphene.^51^ They reported that the force-displacement properties were interpreted within a framework of nonlinear elastic stress–strain response and yields an elastic stiffness of 340 N m^−1^. Such quantities reveal graphene as comparatively the strongest and toughest materials ever investigated. Regardless, the experimental measurements require highly sophisticated facilities. Additionally, it is challenging to synthesize large high-quality 2D monolayers for the analysis. As such, numerical simulations to investigate the mechanical characteristics of 2D materials has become attractive, as evidenced by the blue area in Fig. 2.

**Fig. 3:**
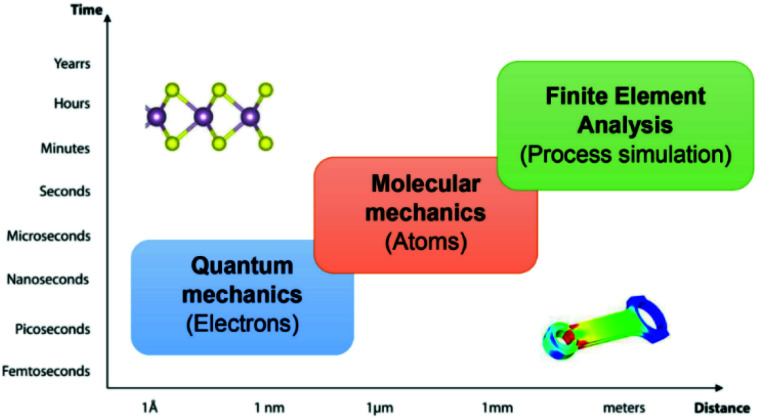
Space and time scale in computational materials science.

Computational materials science has become a rapidly growing multidisciplinary field. Striving to advance computing capabilities to comprehend, understand, and solve the complex problems of functional materials, including their mechanical properties. The mechanical properties are derivable from the linear stress–strain relationship or quadratic energy-strain relationship. Computational approaches with different size and time scales have been developed to explore the microscopic and macroscopic mechanical behaviours of 2D materials. The methods range from the sub-atomic [*e.g.* first-principles density functional theory (DFT)], atomistic level [*e.g.* force field-based classical molecular dynamics (MD)] and macroscopic levels [*e.g.* finite element analysis (FEA)] for the process simulation and engineering design, as illustrated in Fig. 3. In this review, the principles of the numerical simulation of the in-plane mechanical properties of 2D materials are presented. Some recent cases facilitate further discussion about the pros and cons of different methods.

## 2. Methodology

### 2.1 DFT method

The relationship between the in-plane elastic constants and moduli of 2D monolayer materials can be described based on Hooke's law under the in-plane stress condition.1
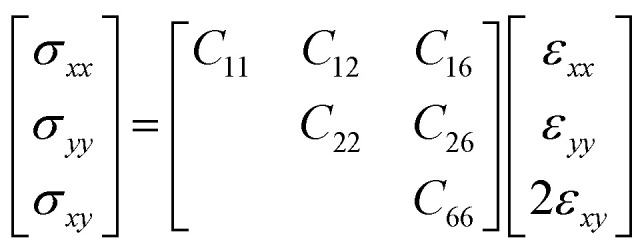
Here *C*_*ij*_ (*i*,*j* = 1,2, and 6) is the in-plane stiffness tensor or in-plane elastic constants, which are equal to the second partial derivative of energy (*E*_s_), with respect to strain (*ε*).

For a first-principles DFT method, the in-plane mechanical properties of 2D materials are calculated through the parabolic dependence of the energy on the elongation.^52–57^ The energy (*E*_s_) can be obtained from the DFT calculations with the different intervals of elongation. Using high-symmetry graphene as an example: the uniaxial strain *ε* was applied along the *x* or *y* direction, which leads to *ε*_*yy*_ = 0 or *ε*_*xx*_ = 0, respectively. Under this condition,2
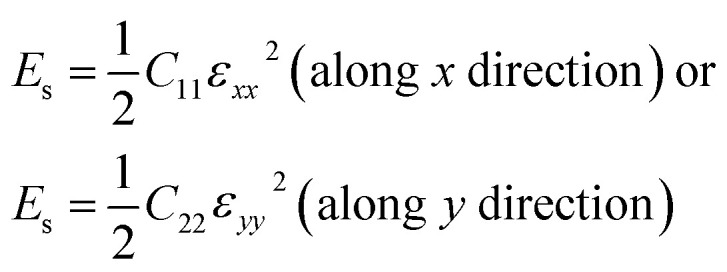
*C*_11_ and *C*_22_ were calculated from the coefficient of the quadratic term by fitting the data of elastic strain energy [*E*_s_(*ε*)] as a function of strain (*ε*). Then, *C*_12_ was calculated based on the equation3
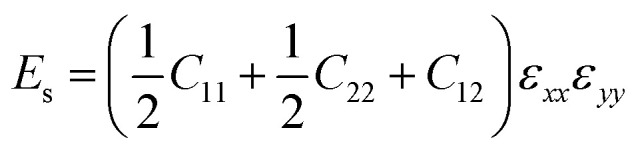


while the equi-biaxial strain is applied. Finally, *C*_66_ was calculated by fitting the second-order polynomial.4
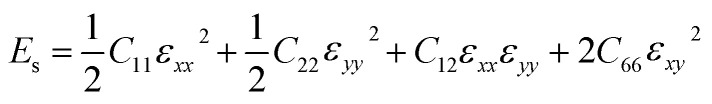


Another method to calculate the in-plane mechanical properties is through the standard approach. In this method, the conventional mechanical properties are first calculated. After that, the multiplication of the conventional stiffness tensors and monolayer thickness will equate to the in-plane stiffness tensors. Here, the thickness of the monolayer needs to be carefully justified. Additionally, the vacuum used to separate the monolayers under the periodic boundary conditions also needs to be thick enough for the converged in-plane stiffness tensors to be retrieved.

The in-plane planar Young's (*Y*^2D^) and shear (*G*^2D^) moduli, together with Poisson's ratio (*ν*^2D^) of 2D monolayer materials, can be derived from the in-plane planar elastic constants as:5
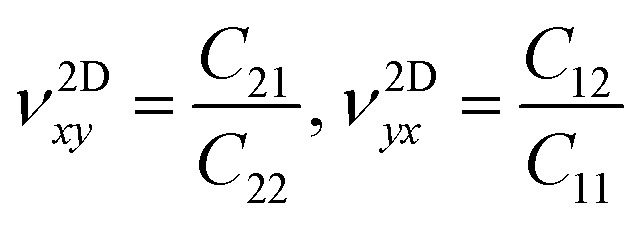
6
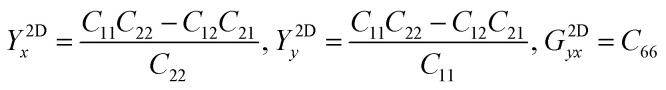


### 2.2 MD method

**Fig. 4:**
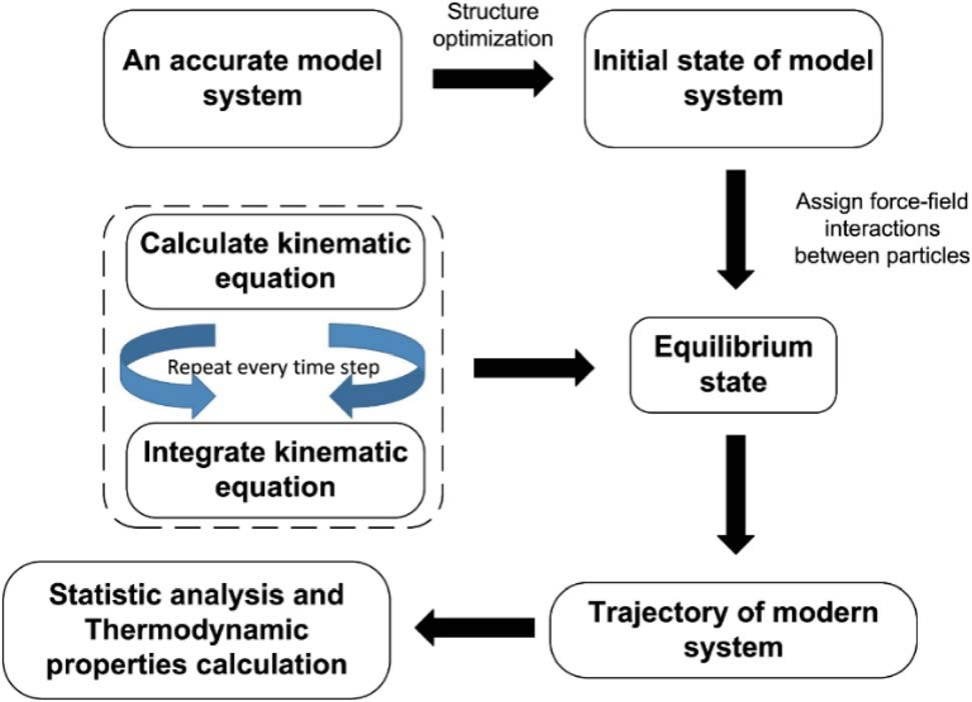
The principle approach of molecular dynamics simulation.

Each atom for MD simulation is subjected to Newton's law of mechanics. The sequential steps to conduct MD simulation can be presented by the flowchart illustrated in Fig. 4.

#### 2.2.1 Stress–strain method

Investigating the in-plane mechanical properties (*e.g.* Young's modulus) of 2D materials required the uniaxial tensile test to obtain the stress–strain relation.^58^ The initial step for the stress calculation is to define the internal pressure tensor *P* for *N* particles in volume *V*:7
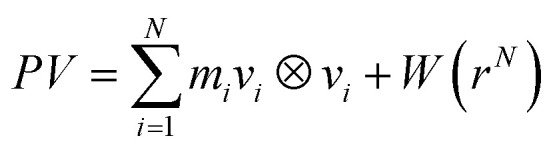
where *m*_*i*_ and *v*_*i*_ are the mass and the velocity of the *i*^th^ particle. ⊗ symbolizes an outer product and *W* is a virial tensor. The interaction between the atoms are accounted for by the force field potential defined as:^59^8
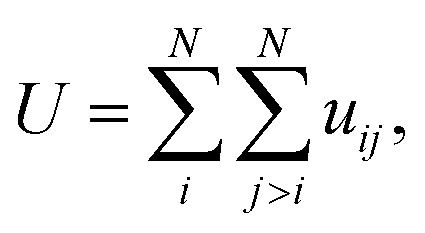


In this equation: *N* is the total number of atoms, *i* and *j* are atomic indices, and *u*_*ij*_ is the empirical potential. *u*_*ij*_ is used to describe the interaction energy between atoms. Each energy term *u*_*ij*_ depends only on a small number (*N*_*k*_) of atoms located at positions *r*_1_,*r*_2_,…,*r*_*N*k_, including atoms *i* and *j*. These atoms can be either in the supercell or in periodic images. Groups are assumingly chosen so that *i* is always in the supercell, which is denoted as **0**. A periodic image of the group belongs to the periodic cell **n**. As such, the potential energy can be expressed as a sum of elements of the primary cell in an unambiguous manner:9
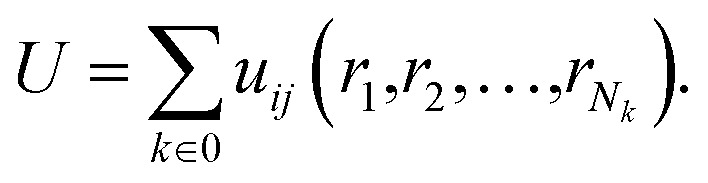


The partial force on a specific atom image can be calculated by deriving this expression with respect to the position *r*_in_ of the periodic image of atom *i* in cell **n**:10
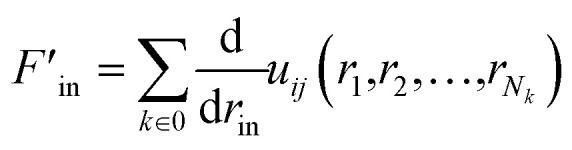


According to the theory of Thompson *et al.*, the virial tensor is:^60^11
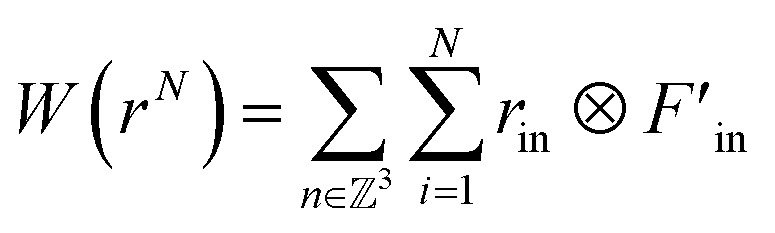


The true mechanical stress tensor is then simply the opposite of the internal pressure tensor:

12 *σ* = −*P*

**Fig. 5:**
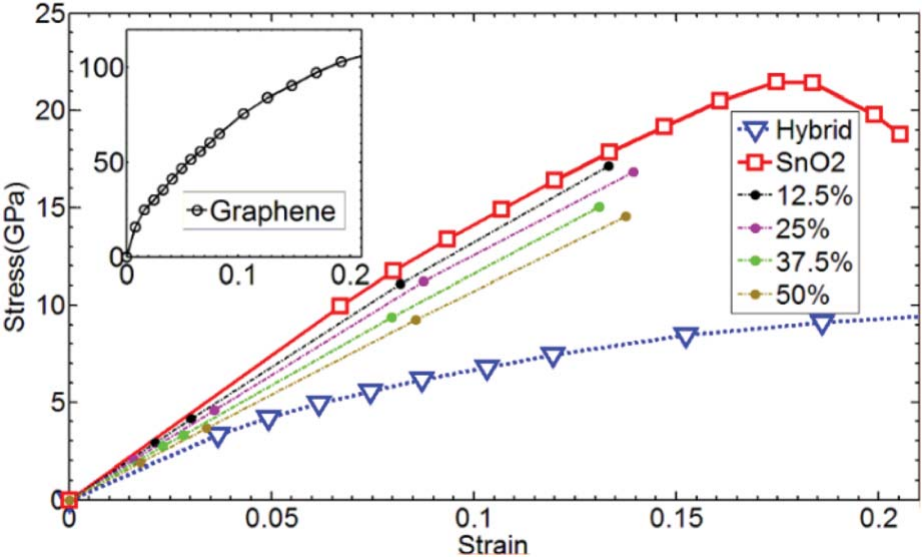
Stress–strain curve of graphene, SnO_2_, and hybrid structures from the ref. 61.

The stress is proportional to the strain when the strain is small, which obeys the general Hooke's law. The Young's modulus can then be calculated because it is the slope of the strain–stress curve, as illustrated in Fig. 5.^61^

#### 2.2.2 Energy-strain method

Identical to the DFT method, the in-plane mechanical properties can be calculated based on the quadratic energy-strain relationship. In the MD simulation, the energy *E*_s_ is calculated using the empirical force field potential based on the atomic configurations of 2D monolayers. The local environment includes the inner state of a molecule, such as the bond length, bond angle, electrostatic forces and van der Waals forces. The total energy of a molecule system is the sum of its kinetic energy and potential energy. Additionally, the force field is a function of potential energy which, can be expressed by the following equations:

13 *E*_total_ = *E*_valvance_ + *E*_nonbond_

14 *E*_valance_ = *E*_bond_ + *E*_angle_ + *E*_torsion_ + *E*_inversion_

**Fig. 6:**
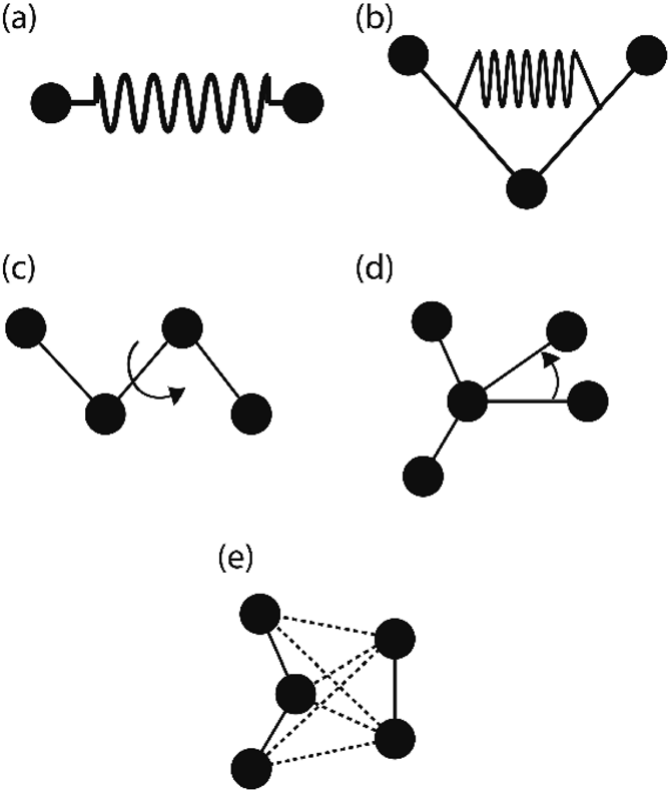
Energy terms, (a) bonding stretching (b) angle bending (c) torsion angle (d) inverted angle (e) nonbonding.

15 *E*_nonbond_ = *E*_vdW_ + *E*_electrostatic_ + *E*_Hbond_

where *E*_total_ refers to total energy of the system, *E*_valance_ is bonding energy, *E*_nonbond_ is non-bonding energy, *E*_bond_ is bonding stretching term, *E*_angle_ is angle bending term, *E*_torsion_ is torsion angle term, *E*_inversion_ is inverted angle term, *E*_vdW_ is energy generated by van der Waals force, *E*_electrostatic_ is energy generated by coulomb electrostatic force and *E*_Hbond_ is hydrogen bond energy. Different types of energy terms are shown in Fig. 6.

### 2.3 FE method

The FE is a numerical method for solving problems, which can be described by partial differential equations or formulated as functional minimization. A domain of interest is represented as an assembly of finite elements. The FEA exhibits two main features. Firstly, the piece-wise approximation of physical fields on finite element provides good precision even with simple approximating functions. Another characterization of the approach is that the locality of the approximation leads to spare equation systems for a discretized problem. Thus, helping to solve problems with a massive number of nodal unknowns.

**Fig. 7:**
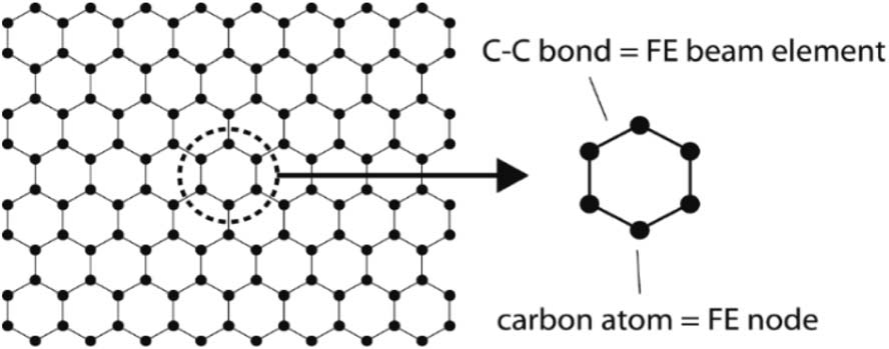
Simulation of a graphene sheet as a space-frame structure.

The FE modelling approach was first utilized in 2003, where the theory of classical structural mechanics was extended into the modelling of nanostructures.^62^ Atoms are bonded together with a corresponding length and angle in a three-dimensional (3D) space. When subjected to loading, materials behave like space frame structures. Thus, the bonds between atoms are considered as connecting load-carrying generalized beam members. Meanwhile, the atoms act as joints of the members, as illustrated in Fig. 7. Structural mechanics analysis is conducted to determine the displacements, strains, and stresses of a structure under given loading conditions. The stiffness matrix approach is one of the elementary instances for solving linear elastic static problems. An additional application is to solve problems that deal with mechanical characteristics such as buckling, plasticity and dynamics. The stiffness matrix *C* consists of the following submatrices:16
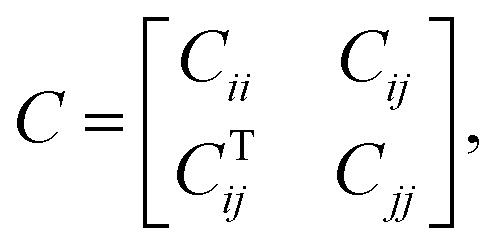
where
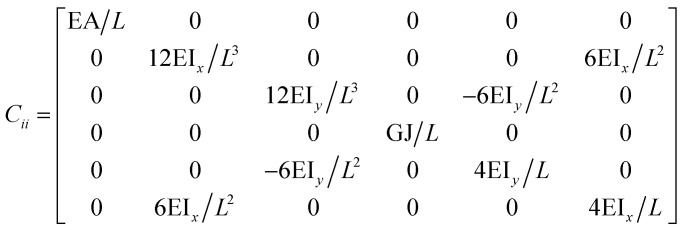

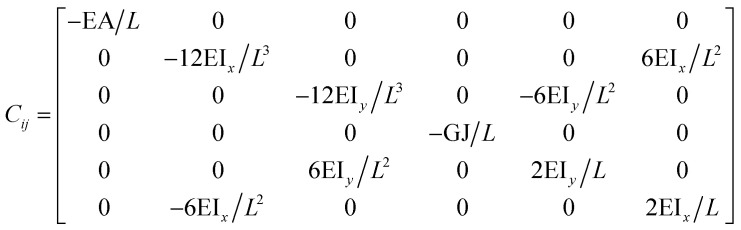

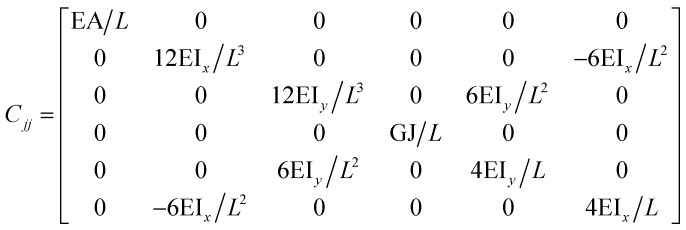


**Fig. 8:**
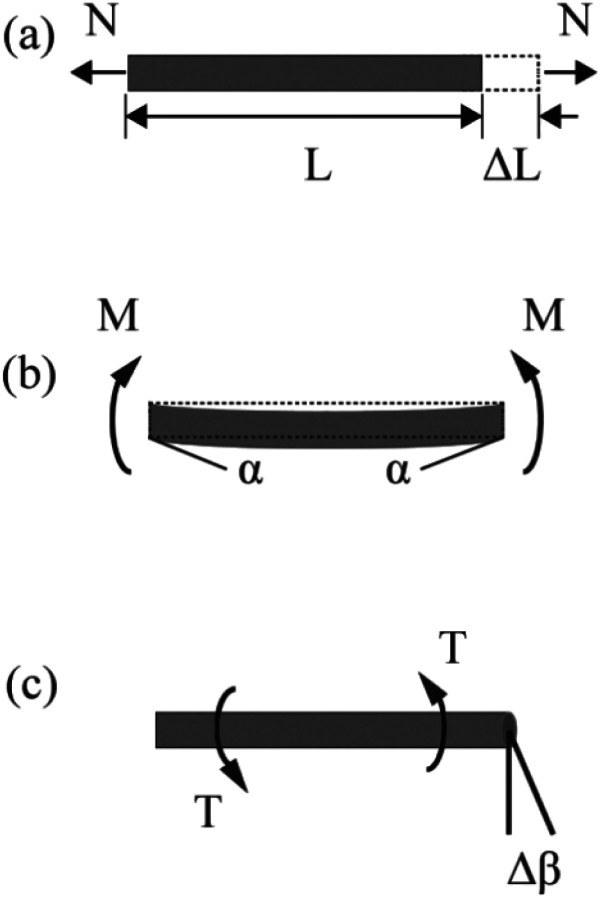
Pure tension, bending, and torsion of an element.

The above elemental stiffness matrices indicate four parameters that are needed including the tensile resistance EA, the torsional stiffness GJ, and the flexural rigidity EI_*x*_ and EI_*y*_. All the while, identifying the length of the element, *L*. The EA, GJ and EI can be determined based on the energy equivalence since the deformation of a space frame results in the changes of strain energies. Based on the theory of classical structural mechanics, the strain energy of a uniform beam of length *L* subjected to pure axial force *N* can be presented as following:17
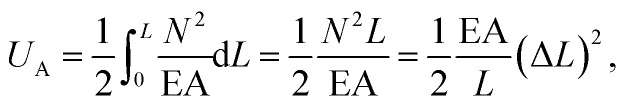
where Δ*L* is the axial stretching deformation, see Fig. 8a.

The strain energy of a uniform beam under pure bending moment *M* is18
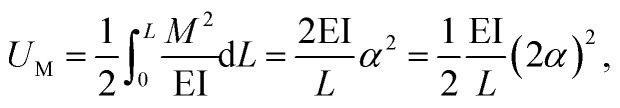
where *α* denotes the rotational angle at the ends of the beam, see Fig. 8b.

The strain energy of a uniform beam under pure torsion *T* is19
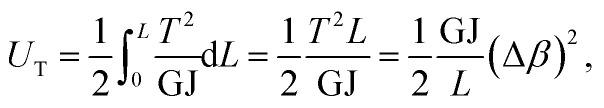
where Δ*β* is the relative rotation between the ends of the beam, see Fig. 8c.

On the other hand, this potential energy between atoms is in the sum of contributions from bond stretch interaction *U*_r_, bond angle bending *U*_θ_, dihedral angle torsion *U*_ϕ_, improper (out-of-plane) torsion *U*_ω_, and a non-bonded van der Waals interaction.^63^ For covalent systems, the main contributions to the total steric energy come from the first four terms, which have included four-body potentials. The harmonic approximation is adequate for describing the energy, considering the assumption of small deformation.^64^ The dihedral angle torsion and the improper torsion are often merged into a single equivalent term for convenience and simplicity's sake, *i.e.*,20
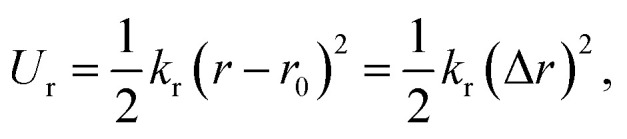
21
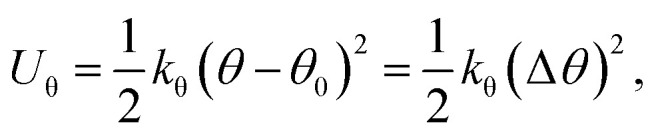
22
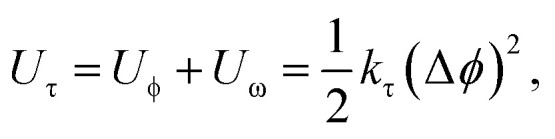
where spring constants *k*_r_, *k*_θ_, and *k*_τ_ are the bond stretching force constant, bond angle bending force constant and torsional resistance respectively. Meanwhile, the symbols Δ*r*, Δ*θ*, Δ*ϕ* represent the bond stretching increment, the bond angle change, and the angle change of bond twisting, respectively.

Here, both *U*_r_ and *U*_A_ represent the stretching energy, both *U*_θ_ and *U*_M_ represent the bending energy, and both *U*_τ_ and *U*_T_ represent the torsional energy. It can also be assumed that the rotation angle 2*α* is equivalent to the total change Δ*θ* of the bond angle, Δ*L* is equivalent to Δ*r*, and Δ*β* is equivalent to Δ*ϕ*. Hence, by comparing eqn (17)–(22), a direct relationship between the structural mechanics parameters EA, EI and GJ can then be deduced from the molecular mechanics parameters *k*_r_, *k*_θ_ and *k*_τ_ as following:23
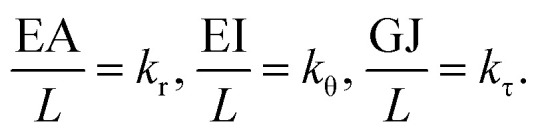


Applying this concept followed by the solution procedure of stiffness matrix method for frame structures, the deformation and related elastic behaviour of 2D materials can be simulated.

## 3. Applications of numerical simulations

### 3.1 DFT studies

#### 3.1.1 Graphene

Investigating the elastic properties (*e.g.* Young's and shear moduli and Poisson's ratio) of 2D materials reveals significant behaviour of the systems and characterizes their structural stability. Various DFT-based studies with differing exchange-correlation functionals have identified the in-plane mechanical properties of pristine graphene.^65–71^ Two different sets of units: (1) GPa and (2) N m^−1^ are often used in the study of 2D monolayers. GPa is for the conventional mechanical properties, and N m^−1^ is for the 2D mechanical modulus, which is converted to the conventional unit through the division of monolayer's thickness. For example, the calculated 2D Young's moduli by Kudin *et al.*^72^ is 345 N m^−1^. Then, its conventional Young's modulus is 1.03 TPa by assuming the think of the graphene is 3.35 Å, which is in good agreement with the measured values. We have used the DFT-D3 method to calculate the in-plane stiffness of graphene. The in-plane elastic constants *C*_11_, *C*_12_ and *C*_66_ are 348, 80 and 133 N m^−1^, respectively. Based on eqn (5) and (6), the calculated in-plane 2D Young's modulus and Poisson's ratio is 329 N m^−1^ and 0.23, respectively. It Implies that the choice of exchange-correlation functional is not sensitive to the calculated 2D mechanical properties of graphene. Likely ascribing to the high in-plane stiffness of graphene, thus directly resulting in a hexagonal lattice and strong carbon–carbon covalent bonds.

#### 3.1.2 TMDs

**Fig. 9:**
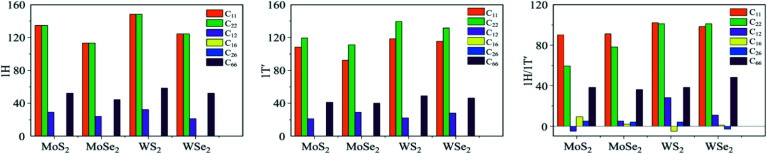
In-plane stiffness tensors *C*_*ij*_ of TMDs with different structural phases from the ref. 83.

Some 2D materials can display several atomic structures that are either sufficiently close in energy to be experimentally observable or stabilized through substrates or doping. For such 2D configurations, the atomic arrangement can significantly affect the physical properties of the system^73–91^ (*e.g.* TMDs). TMDs are one family of well-studied 2D material due to their diverse physical properties in terms of their structural phases.^83,92^ As an instance, molybdenum disulfide (MoS_2_) is reported to be most stable in its 1H semiconducting-like structural phase. However, it has also displayed to be metallic and unstable in its 1T structural phase. Additionally, it exhibits a small bandgap topological insulator in its 1T′ phase.^74,75^ The formation of their lateral heterostructures allows a new degree of flexibility in engineering electronic and optoelectronic devices. Imani Yengejeh *et al.* carried out a comprehensive DFT calculation to study the impact of the structural phases on the mechanical properties of 1H, 1T′ and 1H/1T′ heterostructure phases of different TMD monolayers. Including MoS_2_, molybdenum diselenide (MoSe_2_), tungsten disulfide (WS_2_), and tungsten diselenide (WSe_2_).^83^ Their results reveal that the impact of the structural phase is significant (Fig. 9). The elastic constants of 1H–MoS_2_ were *C*_11_ = 134, *C*_22_ = 134, and *C*_12_ = 29 N m^−1^ which was in good agreement with the ones obtained in the literature (where *C*_11_ = 130, *C*_22_ = 130, and *C*_12_ = 32 N m^−1^, respectively).^93^ The lateral heterostructures had a relatively weak mechanical strength for all the TMD monolayers. As a result, the significant correlation between the mechanical properties of the TMD monolayers and their structural phases can be used to tune their stiffness of the materials.

#### 3.1.3 MXenes

Recently discovered MXenes are a well-known category of chemically active 2D materials often synthesised through the surface functionalisation using terminal groups (*e.g.* –OH, –O, –Cl, –F, or their combinations).^30^ The resulting surface functionalisation led to numerous technological applications, *e.g.* energy storage,^3,94–97^ electromagnetic interference shielding,^98–102^ composite materials,^103–107^ catalysts^108–111^ and sensors.^112–115^ The strain-tunable electrochemical properties of MXenes enable them to be a propitious solution for flexible and stretchable devices.^3,116,117^ Despite over 3000 publications to date on MXenes since their discovery, only 4.9% of the investigations were conducted on the mechanical characterisation of the 2D materials.^30^ On the other hand, a few experimental studies on the mechanical stiffness and strength of MXenes have been published, ascribing to the challenges of the experimental procedures.^118,119^ Therefore, the necessity of conducting more computational studies regarding the mechanical characterisation of MXenes has been increasingly highlighted.

Luo *et al.*^120^ performed a comprehensive DFT-based study to investigate the mechanical properties of the functionalized MXenes. They calculated the elastic constants, free energy, and work function of different MXenes to explore and comprehend the impact of the transition metal and surface functional groups on their characteristics. Apart from some O-termination group exceptions, most of their material models were observed to be dynamically stable. They finally indicated that the Young's moduli for the most investigated MXenes fluctuated from 150 to 400 N m^−1^ and concluded that the functionalized MXenes usually exhibits stronger structural stability. Kazemi *et al.* recently conducted a theoretical investigation to study the mechanical properties of 2D titanium carbide MXene-based materials utilizing DFT calculations.^121^ They discovered that in-plane elastic constants, Young's and shear moduli increase over the thickness of the MXene systems. It further suggests that the improved mechanical properties of the functionalized MXenes. The stronger interaction between the –O terminal group and the Ti_*n*+1_C_*n*_ leads to the stronger mechanical properties of –O functionalized MXenes. Consequently, Ti_4_C_3_O_2_ is the strongest material among all their considered systems. More importantly, all Ti_4_C_3_T_2_ MXenes have the higher in-plane Young's and shear moduli than graphene, which was previously thought to be the strongest known 2D material. Therefore, Ti_4_C_3_T_2_ can be a promising alternative to graphene for applications that need 2D materials with considerably high stiffness and largely resistant to shape change. A similar role for the surface functionalization was also reported by Fu *et al.*^122^ Amongst the five functional groups that they considered, (–F, –Cl, –OH, –H, and –O) the oxygen functionalized Ti_3_C_2_O_2_ possesses the largest adsorption energy, the highest in-plane planar elastic modulus, and the greatest enhancement of strength. Those findings pave the avenue for tuning the mechanical properties of MXene-based materials database by engineering their composition.

#### 3.1.4 Other 2D materials

Up to date, many 2D monolayers have been investigated using the DFT-based energy-strain relationship. Table 2 lists some recent studies for evaluating the mechanical properties of 2D monolayers with different exchange-correlation (XC) functionals.

**Table 2:** Recent investigations for evaluating the mechanical properties of 2D materials using DFT calculations

Reference	2D material	XC functional	Investigated properties	Research summary
Johari and Shenoy^84^	TMDs	GGA-PBE	Mechanical and electrical properties	Performing DFT calculations to evaluate the impact of mechanical strains on the electronic properties of TMDs
Jiao *et al.*^50^	TcS_2_ and TcSe_2_	HSE	Structural, mechanical, electronic, and optical properties	DFT calculations were carried out to study the physical properties of monolayer 2D materials and proposing their potential applications in photovoltaics, photocatalysts, and other nanodevices
Jiao *et al.*^123^	NaSnP	GGA-PBE	Structural, mechanical, electrical, and optical properties	Investigating the physical properties of monolayer NaSnP and suggesting its promising applications in photovoltaic
		HSE		
Lv *et al.*^124^	M_2_Se_3_	GGA-PBE	Mechanical, magnetic, electric, and structural properties	Systematically investigated the physical characteristics of monolayer M_2_Se_3_. Exploring superior mechanical flexibility and negative Poisson's ratio in the studied 2D models
Lü *et al.*^125^	Phosphorene	GGA-PBE	Young's and shear moduli, Poisson's ratio	Exploring the behaviour of phosphorene and its oxides by investigating their mechanical properties
Kazemi *et al.*^121^	MXene	DFT-D3	In-plane planar Young's and shear moduli	Evaluating the characteristics of 2D titanium carbide applying DFT-D3 calculation to predict their mechanical properties of these 2D materials
Imani Yengejeh *et al.*^83^	TMDs	GGA-PBE	Mechanical properties	Investigating the impact of hetero-structure phase on the mechanical properties of TMD monolayers

For example, Aghdasi *et al.* performed DFT calculations to study the in-plane Young's modulus of monolayer phosphorene with and without the adsorption of different atoms, including Li, Mg, O, Al, Pt, Pd, and Mo.^126^ Using the energy-strain method, they found that the in-plane Young's modulus of monolayer phosphorene is anisotropic. Its in-plane Young's modulus along the armchair and zigzag directions are 25.36 and 92.30 N m^−1^, respectively. They also found that the impacts of different adsorbed atoms on the mechanical properties can either be positive or negative. Additionally, the influence of the atomic adsorption on the in-plane Young's modulus of the armchair phosphorene is more remarkable than the zigzag phosphorene. Observing the adsorbed zigzag phosphorene reached the plastic region at smaller strains.

### 3.2 MD simulations

MD based simulation can be a promising approach for investigating the physical and mechanical properties of 2D materials using a relatively large size due to the low computational cost.

#### 3.2.1 Graphene

Both stress–strain and energy-strain methods have been used to study the in-plane mechanical properties of graphene. For the stress–strain method, tensile tests were conducted to identify the Young's modulus, the ultimate tensile strength, and the yield strength. The uniaxial tensile loadings enabled them to obtain the elastic modulus, fracture stress, and fracture strain of graphene with respect to the orientation, sheet size and sheet temperature. Lebedeva *et al.*^127^ evaluated the impact of the different potentials on the in-plane mechanical properties of graphene using the energy-strain method. The Tersoff potential (REBO-1990, REBO-2000, REBO-2002 and AIREBO) and reactive bond-order potentials (LCBOP, PPBE-G, ReaxFF-CHO and ReaxFF-C2013) were used in their study. They uncovered that the impact of the potential was significant. Among all the force fields, LCBOP provided results consistent with the experimental and DFT data. Thus, describing the in-plane deformations of graphene. However, the ReaxFF potentials greatly overestimate the Poisson's ratio. Additionally, the elastic response of all the potentials considered is non-linear already at elongations of 3%. This non-linearity, however, is particularly striking for REBO-2000, AIREBO and REBO2002. It is often responsible for very different results for the Young's modulus and Poisson's ratio across varying intervals of strains and computational approaches.

Javvaji *et al.* used the stress–strain method to investigate the influence of external parameters on the yield properties of graphene.^128^ They also studied the distribution of the stress–strain plots with varying domain sizes, initial crack lengths and lattice orientations. The yield values were observed to drop significantly for small initial crack lengths less than or equal to 0.1 *L*. The yield values were also observed to decrease with the increase in domain size, as the larger specimens offer more spaces for dislocations to initiate. Evidence was given for the combined effect of the domain size, lattice orientation and crack length on the yield values of stress and strain. Gamboa *et al.* conducted a MD simulation to investigate the elastic properties of graphene sheets through implementing the uniaxial tensile tests.^129^ The values of the Young's modulus and Poisson's ratio obtained in their calculation were 730 GPa and 0.39, respectively. Differing significantly from the former estimations and much closer to experimental values. A proposed explanation was that the effect of atomic relaxation leads to a plausible accuracy. They finally observed an extended linear domain in the stress–strain curves, which is relevant to Young's modulus calculations at finite temperature.

Anastasi *et al.* conducted an intriguing investigation on the mechanical properties of pristine and nano-porous graphene using MD simulations.^130^ They studied the fracture behaviour, and the temperature dependence of the 2D material models. Additionally, they investigated the random and uniformly distributed vacancy defects. They discovered that higher temperatures tend to significantly decrease the fracture stress and strain almost linearly with temperature. The elastic modulus was, however, only affected at system temperatures higher than approximately 900 K. Thus, concluding that the fracture stress decreases substantially inversely proportional to the defect density. Their latter attempt was made for potential applications which require non-pristine sheets such as filtration membranes.

#### 3.2.2 TMDs

A MD simulation with the stress–strain method was performed by Ying *et al.*^131^ to study the mechanical behaviour of synthesized ternary TMDs under two loading conditions: the uniaxial tension along the armchair and zigzag directions and the biaxial tension along both directions. They examined the impact of loading direction, the temperature on the Young's modulus and also the fracture behaviour of their investigated TMD nanosheets. The force interactions between atoms in their system were described by the Stillinger–Weber (SW) potential, which parameters were achieved by fitting the SW potential to an experimentally obtained phonon spectrum. Based on their MD results, the in-plane Young's modulus of monolayer MoS_2*x*_Te_2(1−*x*)_ was slightly affected when *x* is in the range of 0–0.4. Yet, ostensibly increased when *x* is larger than 0.4, which demonstrated that the Young's modulus of MoTe_2_ is insensitive to doping S atoms inside them. Furthermore, it shows that the Young's modulus of MoS_2_ nanosheets is very sensitive to the doping Te atoms inside the nanosheets. Moreover, MoS_2*x*_Te_2(1−*x*)_ was found to possess a ductile fracture feature. Their Young's modulus and ultimate strength decreased when the temperature became higher, which was ascribed to the temperature-induced softening effect.

Zhao *et al.* conducted a MD study to evaluate the temperature-dependent mechanical properties of monolayer MoS_2_.^132^ Their computational results stated that the mechanical characterizations of the material model vary with temperature. More precisely, the Young's modulus, maximum loading strain, and maximum load stress decrease with the rise in temperature from 4.2 K to 500 K. They also discussed that the tendency of the maximum loading strain with the different temperature is opposite to that of metal materials which are caused by the short-range SW potentials among the TMD atoms. Despite holding the high computational efficiency, the mathematical form of the SW potential may not be suitable for the atomic-thick planar structures, such as graphene and b-BN, as such potentials are less probably to resist the bending motion of these real planar crystals.^133–135^

Jiang performed a MD simulation to study the misfit strain-induced buckling of the TMDs lateral heterostructures, such as MoS_2_–WSe_2_ and MoS_2_–MoTe_2_.^136^ He also utilized the SW potential to describe the interatomic interactions for each TMD and the coupling between different TMDs. Computational results indicated that misfit strain presumably causes buckling of the TMD with larger lattice constants in the lateral heterostructure, due to the atomic-thick nature of TMDs. These results raise many mechanical challenges for the structural stability of the low-dimensional materials.

#### 3.2.3 Other 2D materials

Due to the limited availability of force field potentials, the studies on other 2D materials are few. Table 3 summarizes some recent studies for evaluating the mechanical properties of 2D materials using MD methods.

**Table 3:** Recent studies evaluating the mechanical properties of 2D materials using MD approaches

Reference	2D material	Investigated properties	Research summary
Jhon *et al.*^137^	Functionalized MXenes	Tensile mechanical response	Studying the surface termination effect on the mechanical response of the MXenes using MD simulation. Exploring the tensile variations of the MXenes under the impact of surface vacancies
Chang *et al.*^138^	Phosphorene	Young's modulus	Examining the mechanical properties of black and blue phosphorene. The results indicate that the temperature has weak impact on the Young's modulus of the structures
Vijayaraghavan and Zhang^139^	Boron nitride–carbon nanosheet	Mechanical properties	Investigating the tensile characteristics of single layer BN–C nanosheets. It was shown that the BN–C nanosheet with parallel arrangement exhibits slightly improved mechanical resistance than the BN–C nanosheet with series arrangement
Hou and Yang^58^	Graphene oxides	Tensile properties	Carrying out tensile test to investigate the mechanical properties of graphene oxide sheets
Javvaji *et al.*^128^	Graphene	Yield stress and strain	Investigating the combined effect of domain size, crack length, and lattice orientation on the mechanical properties of graphene
Anastasi *et al.*^130^	Graphene	Mechanical properties and fracture behaviour	Carrying out uniaxial tensile loading to investigate the mechanical properties of pristine and nanoporous graphene. An increase in system temperature results in a significant reduction in the fracture stress and strain
Siriwardane *et al.*^140^	Sulfur-functionalized MXenes	Structural, stability, and ion dynamic properties	Performing computational calculations at 400 K and bond-length analysis to study the physical properties of functionalized MXenes
Cellini *et al.*^141^	Diamond boron-nitride	Mechanical properties and pressure induce phase transition	Investigating the mechanical properties and pressure-induced formation of 2D diamond boron nitride. The results show a metastable local rearrangement of the h-BN atoms into diamond crystal clusters when increasing the indentation pressure

One recent MD simulation example conducted by Yang *et al.* investigated the temperature-dependent stress–strain relations of monolayer black phosphorus while considering the impact of temperature changes.^142^ The Young's and shear moduli under uniaxial tension at different temperatures were investigated. The values of the Young's and shear moduli ranged from 24 to 19.2 GPa and 25.4 to 20 GPa, respectively. Observations showed that for a given temperature, the Young's moduli along the zigzag direction are more than four times higher than those along the armchair direction, which matches the DFT conclusion.^126^ Their predicted strength and moduli were in good agreement with the available experimental data.

### 3.3 FEM simulations

Due to its significant advantages, the FEM approach has become increasingly popular and a promising alternative for evaluating the macroscopic mechanical properties of low-dimensional materials for a wide range of emerging applications where high conductivity, mechanical strength, high surface area, and high yield strain are required.^143,144^ FEM's features enable researchers to work on a larger scale of the material models, which consists of numerous atoms in their structural configurations.

#### 3.3.1 Graphene

Scarpa *et al.* proposed a FE model and an approach based on cellular material mechanics theory to study the in-plane linear elastic properties of the monolayer graphene sheets. In their material model,^145^ the C–C bonds were represented by equivalent mechanical beams having full stretching, hinging, bending and deep shear beam deformation mechanisms. Finally, using equivalent mechanical C–C bond characteristics, the in-plane Young's, shear moduli and the Poisson's ratio were derived.

FEA is also strongly beneficial for conducting the simulations to explore the mechanical properties of defective low-dimensional systems. Recently, Nakanishi *et al.* investigated the macroscopic mechanical properties of hollow-wall graphene gyroids by performing indentation tests with suitable interpretation by FE simulations.^146^ They carried out two sets of investigations. Firstly, they used periodic cell calculations to explore the mechanical properties of their systems in terms of the relative density and cell wall characterization of the lattice. Then, the indentation simulations of a continuum with the effective properties of the gyroid were performed. They finally concluded that hollow-wall graphene gyroids combine size-dependent mechanical and electrical properties with a topology of high structural efficiency.

#### 3.3.2 TMDs

Recently, Li *et al.* combined the AFM and FEM to investigate the in-plane Young's modulus of 1H/2H MoS_2_ monolayer and bilayer.^147^ The bimodal AFM is first employed to figure out the effective spring constant between the microscope tip and sample. After that, the FEM is developed to quantitatively ascertain the effect of substrate stiffness on deformation. Using this combined method, they calculated the conventional in-plane Young's modulus of monolayer MoS_2_ of 265 GPa after removing the impact of the substrate. When assuming a 1H MoS_2_ monolayer thickness of 6.15 Å, the corresponding 2D in-plane Young's modulus is 163 N m^−1^ and comparably close to the DFT value of 165 N m^−1^.^148^ Therefore, this combined method provides a convenient approach to calculate the in-plane Young's modulus of 2D materials on a substrate.

#### 3.3.3 Other 2D materials

Up to date, the FE studies on the in-plane mechanical properties of other 2D materials are scarce due to the restriction of the available spring constants. Table 4 summarizes the most recent studies for evaluating the mechanical properties of 2D materials using the FEM.

**Table 4:** Recent studies evaluating the mechanical properties of 2D materials using FE approaches

Reference	2D material	Approach	Investigated properties	Research summary
Khandoker *et al.*^149^	Graphene	FEM	Young's modulus, shear modulus, and Poisson's ratio	Investigating the mechanical properties of graphene using atomistic modeling and continuum approaches on mono- and double-layer graphene. The number of layers affects the Poisson's ratio but not the Young's and shear moduli
Damascento *et al.*^150^	Graphene	FEM	Tensile and fracture strength	Conducting an atomistic simulation of FEM to investigate the impact of structural defects on the mechanical properties of graphene
Li *et al.*^147^	MoS_2_	FEM	Young's modulus	Demonstrating an approach to map the in-plane Young's modulus of single- and double-layer MoS_2_. The elasticity of both systems cannot be differentiated
Zhang *et al.*^151^	Graphene	FEM	Mechanical properties	Modifying the properties of Sn–Cu–Ni solder joint used for solar cells by exploring their mechanical characteristics. Applying FEM to calculate the stress–strain curve
Nakanishi *et al.*^146^	Hollow-wall graphene gyroids	FEM	Elastic modulus and yield strength	Evaluating the macroscopic mechanical properties of solid-wall nickel gyroids and hollow-wall graphene gyroids using nano-indentation testing with a suitable interpretation by FE simulation
Imani Yengejeh *et al.*^152^	Graphene	FEM	Natural frequency	Performing a numerical investigation to study the mechanical properties of topologically defective and functionalized graphene sheets. Reporting the reduction of natural frequencies of the material models due to the presence of the atomic defects

### 3.4. Discussion

Overall, numerical modelling approaches with different size and time scales have been developed for evaluating the in-plane mechanical characteristics of 2D monolayers.

The in-plane elastic properties of some of the most studied 2D materials are presented in Table 5.^51,83,121,147–149,153–157^ Basically, in-plane mechanical properties (such as Young's modulus, shear modulus, hardness, and Poisson's ratio) can be calculated through the linear stress–strain or quadratic energy-strain relationships. DFT methods are independent of any empirical parameters, which provide the highest accuracy and flexibility, as evidenced by the data listed in Table 5. The DFT in-plane Young's modulus and Poisson's ratio of graphene and MoS_2_ are almost identical to the measured values. There is a considerably large difference in the MXene system. Because materials used for experimental measurements have hybrid terminal groups, this consequently leads to the theory-experiment discrepancy. The simulation quality of MD simulation is greatly affected by the force fields used in the study, as evidenced by Lebedeva *et al.* on graphene.^127^Table 5 lists one of the best MD results, which are also close to the experimental data. These results are caused by the potentials for graphene and TMDs being purposely optimized. Up to date, graphene is the most studied system *via* the FEA method. However, its calculated in-plane mechanical properties are far from the experiments. For example, the calculated Poisson's ratio of graphene is about four times higher than the DFT and experimental values.

**Table 5:** In plane Young's modulus (*Y*^2D^), shear modulus (*G*^2D^) and Poisson's ratio (*ν*^2D^) of 2D materials obtained by different computational approaches and experimental measurements

2D Material	Method	Procedure	*Y* ^2D^ (N m^−1^)	*G* ^2D^ (N m^−1^)	*ν* ^2D^	Ref.
Graphene	DFT	Energy-strain	345	150	0.149	Wei *et al.*^153^
	MD	Stress–strain	320	150	0.22	Kalosakas *et al.*^154^
	FEA	—	272–323	85–153	0.7–0.8	Khandoker *et al.*^149^
	Exp.	AFM	340 ± 50		0.165	Lee *et al.*^51^
MoS_2_	DFT	Energy-strain	127.7	52	0.22	Imani Yengejeh *et al.*^83^
	MD	Stress–strain	149.42	—	—	Mortazavi *et al.*^155^
	FEA	—	163	—	—	Li *et al.*^147^
	Exp.	AFM	120	—	0.29	Cooper *et al.*^148^
Ti_3_C_2_O_2_	DFT	Energy-strain	366–372	145	0.258	Kazemi and Wang^121^
	MD	Stress–strain	378.3	—	0.29	Plummer *et al.*^156^
	FEA	—	—	—	—	—
	Exp.	AFM (Ti_3_C_2_T_*x*_)	326 ± 29	—	—	Li *et al.*^157^

In addition, the first principles DFT method does not require any pre-set parameters. Its simulations can study chemical reactions involving charge transfer, bond formation and cleavage with high accuracy. Thus, DFT-based simulations provide greater flexibility for calculating the in-plane mechanical properties of 2D materials. As a comparison, only limited force fields are available for most of the 2D materials beyond graphene. The parametrization of the force field either derived from the experimental data or first-principles DFT results is a very time-consuming task. As such, MD-based methods cannot be widely used. The FEA simulation face the same problem because its quality is based on the spring constants, which have the same issues related to the availability and quality.

On the other hand, the force-field-based classical MD methods use empirical interatomic potentials to describe their interaction energies. Thus, significantly reducing the computational time using low cost. Consequently, the MD method can assess the mechanical properties of large 2D materials. Additionally, the impact of temperature on the mechanical properties can also be evaluated. The FEM has the lowest computational cost. Most of the FEM computations can be conducted using desktop computers. Consequently, it can be used to investigate some macroscopic mechanical properties.

To obtain the holistic picture of in-plane mechanical properties of 2D materials, a combination of different computational methods is a plausible approach by taking advantage of each one's strong features. So far, there have been some attempts to combine the computational methods and propose a refined technique to investigate the mechanical properties of the nanostructures. Imani Yengejeh *et al.* proposed a refined FEA to evaluate the vibrational properties of the defective CNTs and their modifications.^158^ They implemented the well-established FEA for the perfect material models. For appraising the properties of the defective structure, the accurate DFT structural relaxation was used as FEA cannot consider the rearrangement of the atoms after the introduction of the defect (*e.g.* vacancy defect). Their obtained results were in good agreement with the experimental findings, which suggested that such a combined multiscale modelling approach is feasible for investigating the mechanical characteristics of the 2D materials.

## 4. Summary and outlook

Over the past few decades, the family of low-dimensional materials has rapidly grown beyond graphene. Many novel 2D materials including, TMDs, MXene and phosphorene, have become significantly important. The remarkable properties of 2D materials pave a bright future for basic research and large-scale industrial applications. Due to several limitations of the experimental measurements, numerical simulation becomes a promising alternative to evaluate the in-plane mechanical properties of these 2D monolayers.

This review described some widely used computational approaches for investigating the in-plane mechanical properties of 2D material. Their features are discussed using some recent study results as examples. DFT-based computation is the most widely used approach to understand the in-plane mechanical properties of 2D materials since their results are reliable. All 2D monolayers can theoretically be investigated using the DFT method after their atomic configurations are correctly built. The DFT results can be used to guide their design and screen for 2D materials at the atomic level to achieve desired mechanical properties. A major disadvantage of the first-principles calculations is that only a few thousand atoms can be simulated. Consequently, seriously limiting the direct comparisons to experimental studies. In experiments, the measurements typically occur on the micrometre length scale. For such larger sizes, classical MD and FEM simulations are desirable. The minuscule computational costs of atomistic simulations using empirical force field potentials and spring constants enable the mechanical properties of some 2D systems to be predicted. Basically, MD simulation is one of the molecular simulation techniques which refer to a set of approaches a conducting computational experiment on molecule models. This simulation method can be classified into two major categories: microscale defined in the range of 0.1–10 nm and mesoscale defined from 10 nm to 100 nm. However, the key ingredient for MD and FEM simulations is an empirical interatomic potential and spring constants, which determines the availability and accuracy of MD and FEM simulations. Since different computational approaches exhibit their advantages and disadvantages, the most appropriate computational technique can be selected in terms of the type of mechanical properties of the 2D material, the required accuracy of the results, and the features of the applied method.

Finally, the development of novel technologies becomes essential to generate a comprehensive understanding of the in-plane mechanical properties of the 2D materials for their applications based on strain engineering. Several promising methods are discussed below:

(1) A combining approach may be an excellent solution to describe a wide range of 2D materials properties as it would enable the incorporation of positive features from each method. For example, the machine learning method has been used to develop the force fields from the DFT results, which can greatly expand the application area of the MD and FEM simulations.^159–162^

(2) The combination of the experimental measurements and the numerical simulations also holds great potentials. As suggested by the recent study, the experimental measurements can be used to provide accurate spring constants, which can enable the reliable in-plane Young's modules using the low-cost FEA approach.^163,164^

(3) Machine learning technology can also be used to accelerate the advancement of this area. The DFT-based computational data can be used to build the database. The ensemble learning system can be built using a diverse set of base regressors/classifiers. Construction is done through both traditional learning algorithms (such as support vector machine, decision tree, kernel ridge regression, Gaussian mixture regression) and deep learning algorithms to identify the relationship between the atomic fingerprints and their in-plane mechanical properties.^165,166^

## Conflicts of interest

There are no conflicts to declare.

## Supplementary Material
